# Role of tumor suppressor genes in the cancer-associated reprogramming of human induced pluripotent stem cells

**DOI:** 10.1186/scrt447

**Published:** 2014-04-28

**Authors:** Ying-Chu Lin, Yoshinobu Murayama, Koichiro Hashimoto, Yukio Nakamura, Chang-Shin Lin, Kazunari K Yokoyama, Shigeo Saito

**Affiliations:** 1 School of Dentistry, College of Dental Medicine, Kaohsiung Medical University, 100 Shin-Chuan 1st Road, Kaohsiung 807, Taiwan; 2 Cancer Center, Kaohsiung Medical University Hospital, 100, Tzyou 1st Road, Kaohsiung 807, Taiwan; 3 College of Engineering, Nihon University, Koriyama, Fukushima 963-8642, Japan; 4 Department of Zootechnical Science, Tokyo University of Agriculture, Atsugi, Kanagawa 243-0034, Japan; 5 Cell Engineering Division, RIKEN BioResource Center, Tsukuba, Ibaraki 305-0074, Japan; 6 Graduate Institute of Medicine, College of Medicine, Kaohsiung Medical University, 100 Shin-Chuan 1st Road, Kaohsiung 807, Taiwan; 7 Department of Biological Science, National Sun Yat-sen University, 70 Lien-hai Road, Kaohsiung 804, Taiwan; 8 Saito Laboratory of Cell Technology, Yaita, Tochigi 329-1571, Japan; 9 School of Science and Engineering, Teikyo University, Utsunomiya, Tochigi 320-8551, Japan

## Abstract

Because of their pluripotent characteristics, human induced pluripotent stem cells (iPSCs) possess great potential for therapeutic application and for the study of degenerative disorders. These cells are generated from normal somatic cells, multipotent stem cells, or cancer cells. They express embryonic stem cell markers, such as OCT4, SOX2, NANOG, SSEA-3, SSEA-4, and REX1, and can differentiate into all adult tissue types, both *in vitro* and *in vivo.* However, some of the pluripotency-promoting factors have been implicated in tumorigenesis. Here, we describe the merits of tumor suppresser genes as reprogramming factors for the generation of iPSCs without tumorigenic activity. The initial step of reprogramming is induction of the exogenous pluripotent factors to generate the oxidative stress that leads to senescence by DNA damage and metabolic stresses, thus inducing the expression of tumor suppressor genes such as *p21*^*CIP1*^ and *p16*^*INK4a*^ through the activation of p53 to be the pre-induced pluripotent stem cells (pre-iPSCs). The later stage includes overcoming the barrier of reprogramming-induced senescence or cell-cycle arrest by shutting off the function of these tumor suppressor genes, followed by the induction of endogenous stemness genes for the full commitment of iPSCs (full-iPSCs). Thus, the reactive oxygen species (ROS) produced by oxidative stress might be critical for the induction of endogenous reprogramming-factor genes via epigenetic changes or antioxidant reactions. We also discuss the critical role of tumor suppressor genes in the evaluation of the tumorigenicity of human cancer cell-derived pluripotent stem cells, and describe how to overcome their tumorigenic properties for application in stem cell therapy in the field of regenerative medicine.

## Introduction

### Reprogramming of induced pluripotent stem cells and tumorigenic properties

Stem cells with the capacity to differentiate into all adult tissue types can be derived from the inner cell mass of the mouse blastocyst [1]. These embryonic stem cells (ESCs) are unique resources for the research of cell development and differentiation, with the ultimate aim of repairing damaged tissues and organs in humans. The reprogramming of differentiated mammalian somatic cells into an undifferentiated pluripotent state was first demonstrated by the birth of viable young sheep after nuclear transfer of adult somatic cells into unfertilized enucleated oocytes [2]. However, the approaches used to obtain pluripotency in humans, such as the nuclear transfer of somatic cells or the fusion of somatic cells with ESCs, have always been associated with ethical concerns that interfere with the application of these types of cells in basic research and clinical therapy. The successful reprogramming of mouse somatic cells to induced pluripotent stem cells (iPSCs) by the enforced expression of pluripotency factors [3] has paved the way for autologous cell-based therapeutic applications and the study of degenerative disorders. Subsequent reports have demonstrated that iPSCs are highly similar to ESCs when tested using a serial set of assays [4-6]. The use of such cells can circumvent the ethical concerns described above.

The core ESC regulatory circuitry involves OCT4, SOX2, and NANOG, which regulate their own expression and the expression or suppression of other factors involved in self-renewal, pluripotency, and dedifferentiation [7-10]. Recently, two reports showed that TFCP2L1 is another critical factor for nuclear reprogramming [11,12]. Several studies have shown that the activation of the Wnt pathway can cause ESCs to remain pluripotent [13-17]. In contrast, other studies demonstrated that the Wnt pathway controls the differentiation of ESCs and the terminal differentiation of postmitotic cells [18,19]. Furthermore, another group observed that OCT4 regulates pluripotency via nuclear β-catenin degradation, thereby antagonizing Wnt-β-catenin signaling, and that the downregulation of OCT4 increases β-catenin protein levels, thus enhancing Wnt signaling and initiating the differentiation of ESCs [20]. Some of the pluripotency factors used to generate iPSCs have been implicated in tumorigenesis, indicating that reprogramming and cellular transformation might occur via related pathways [8,21-23]. Interestingly, the inhibition of the tumor suppressor p53 (the product of the human *TP53* and mouse *Trp53* genes) enhances the reprogramming of fibroblasts into iPSCs [24] and can generate transformed cancer stem cells from differentiated cells [25]. The efficacy of the nuclear reprogramming of cancer cells with mutated p53 or deleted p53 is increased to generate iPSCs; however, the frequency of tumorigenesis is also clearly increased in these reprogramming cancer stem cells [26]. Thus, none of the traditional models incorporates the possibility of tumor-associated cellular reprogramming and the plasticity associated with the loss of p53 function. Therefore, the tumorigenicity risk associated with these stem cells must be removed before the achievements observed in basic research can be safely translated into clinical applications.

In this review, we summarize the connection between tumor suppressor genes (to avoid the emergence of tumor cells) and full reprogramming to iPSCs. We address the question of whether cancer-cell-specific iPSCs are equivalent to other types of stem cells, such as fully committed iPSCs (full-iPSCs), from the point of view of overcoming their tumorigenic properties.

### Role of gatekeeping tumor suppressors in stem cells

Stem cell genomes must be rigorously ‘guarded’ throughout each developmental stage because such cells expand periodically to enable tissue repair and replacement. Thus, as faithful genomic duplication over a lifetime is restricted to minimize the accumulation of oncogenic lesions during such expansions, inadequate genomic stability control would be especially deleterious in ESCs because they are the progenitors of all adult organ systems. Gatekeeping tumor suppressors, such as p16^INK4a^, p14^ARF^, and p53, negatively regulate cellular proliferation and survival [27]. These gene products were first discovered by virtue of their role in cancer, but probably evolved to regulate homeostasis in normal tissues by regulating the proliferation and survival of normal cells. Gatekeeping tumor suppressors tend to negatively regulate stem cell function [28] and regulate stem cell aging because their expression and/or function increase with age [29-31]. Elevated p53 expression or constitutive p53 activation can deplete stem cells [32], causing premature aging, and shorten life-span despite reducing cancer incidence [33-35]. These effects in mice also appear to reflect similar functions in humans because a polymorphism in p53 that reduces p53 function increases cancer incidence and life-span in humans [36]. This suggests that increased p53 activity protects against cancer but can promote aging and shorten life span, at least when a certain threshold of activity is reached. The functions of the p16^INK4a^, p14^ARF^, and p53 tumor suppressors depend on expression level and context, thus promoting the maintenance of mitotically active cells in some contexts, while promoting cell death or senescence in other contexts. For example, p53 promotes the maintenance of genome integrity [37] and promotes tissue generation in ATR mutant mice by promoting DNA repair and/or by promoting the death of cells with DNA damage [38]; however, in response to oncogenic stimuli or telomere attrition, p53 depletes stem cells [32,39]. Overall, gatekeeping tumor suppressors have pleiotropic functions that promote stem cell functions in some ways and negatively regulate them in other ways, with complex and context-dependent consequences for aging.

### Deficiency of p53 and stemness characteristics

Although p53 mutation and pathway inactivation are found in the majority of tumors, they appear to be especially concentrated among tumors that exhibit plasticity and loss of differentiation characteristics [40-42]. Selection for p53 functional inactivation during cancer progression has typically been attributed to the survival benefits that result from reduced apoptosis, cell cycle arrest, and increased opportunities for cellular evolution afforded by genomic instability. In light of the above discussion, however, it is also possible that p53 loss destabilizes the differentiated state and enables reversion to a more stem-like state. It is well known that the inhibition of the p53 pathway increases the apparent efficiency of iPSC generation dramatically [43-47]. The downregulation of genes that contribute to cell-cycle arrest or apoptosis also increases reprogramming. For example, although a mutation in MDMX reduced p53 activity by only two-fold at baseline, it increased reprogramming efficiency dramatically [45]. These results have several important implications. First, subtle changes in p53 activity are all that is required to increase the probability of reprogramming. Second, reprogramming is limited by a variety of p53-induced protective pathways, including, but not limited to, those involved in cell-cycle arrest, senescence, and apoptosis. Third, through its ability to inhibit cell-cycle progression, p53 provides a potent barrier to the acquisition of the dedifferentiation involved in iPSC formation. Understanding of the mechanisms via which p53 limits reprogramming is complicated by the various methods used for the introduction of the reprogramming factors, as well as by the expression levels of these factors. In terms of stress induction, however, all these commitments are similar in the case of the induction of oxidative stress and production of reactive oxygen species (ROS).

### Roles of reactive oxygen species and tumor suppressor genes during reprogramming

The cellular damage caused by free radicals may generate ROS as a consequence of oxidative phosphorylation in the mitochondrial electron transport chain [48]. ROS, such as superoxide and hydroxyl radical, are highly reactive and can damage mitochondrial and nuclear DNA, as well as proteins and lipids, by modifying them chemically. Nuclear reprogramming induced by Yamanaka factors involves extensive chromatin remodeling and resets the epigenetic program to generate iPSCs [49]. This conventional iPSC technique using virus-mediated gene transfer is now a common method to deliver reprogramming factors [50]. In fact, the virus infection-induced immune response, like innate immunity, can result in accumulation of ROS [51,52]. Alternative reprogramming methods without virus infection might be useful to increase the survival rate of iPSCs due to less ROS production. Stem cells appear to be particularly sensitive to elevated ROS levels. Increased ROS levels resulting from metabolic changes in iPSCs may hinder the survival of reprogrammed cells, as suggested by observations of iPSC-generation under hypoxic conditions [53,54]. In addition, mitochondrial functions are also repressed in iPSCs or human ESCs [55], suggesting that ROS generation by reprogramming factors is unfavorable to the generation of iPSCs. Vitamin C has been reported to be an effective chemical to boost iPSC generation. Treatment with vitamin C reduced p53/p21 levels, which are the main barrier to successful reprogramming [56]. Wang *et al*. [57] found that the histone demethylases Jhdm1a/1b are the direct downstream effectors of vitamin C, in addition to antioxidant activity. Jhdm1b promotes cell-cycle progression and suppresses senescence by repressing the INK4a/ARF locus during reprogramming. Furthermore, inhibition of the mammalian target of rapamycin (mTOR) pathway by rapamycin, PP242, or the insulin/insulin growth factor-1 (IGF-1) signaling pathway notably enhances the efficiency of reprogramming [58]. Based on the concept that reprogramming is a stressful process that activates apoptosis and cellular senescence, it was shown that targeting the mTOR pathway alleviates the senescence imposed by the DNA damage response [59].

**Figure 1:**
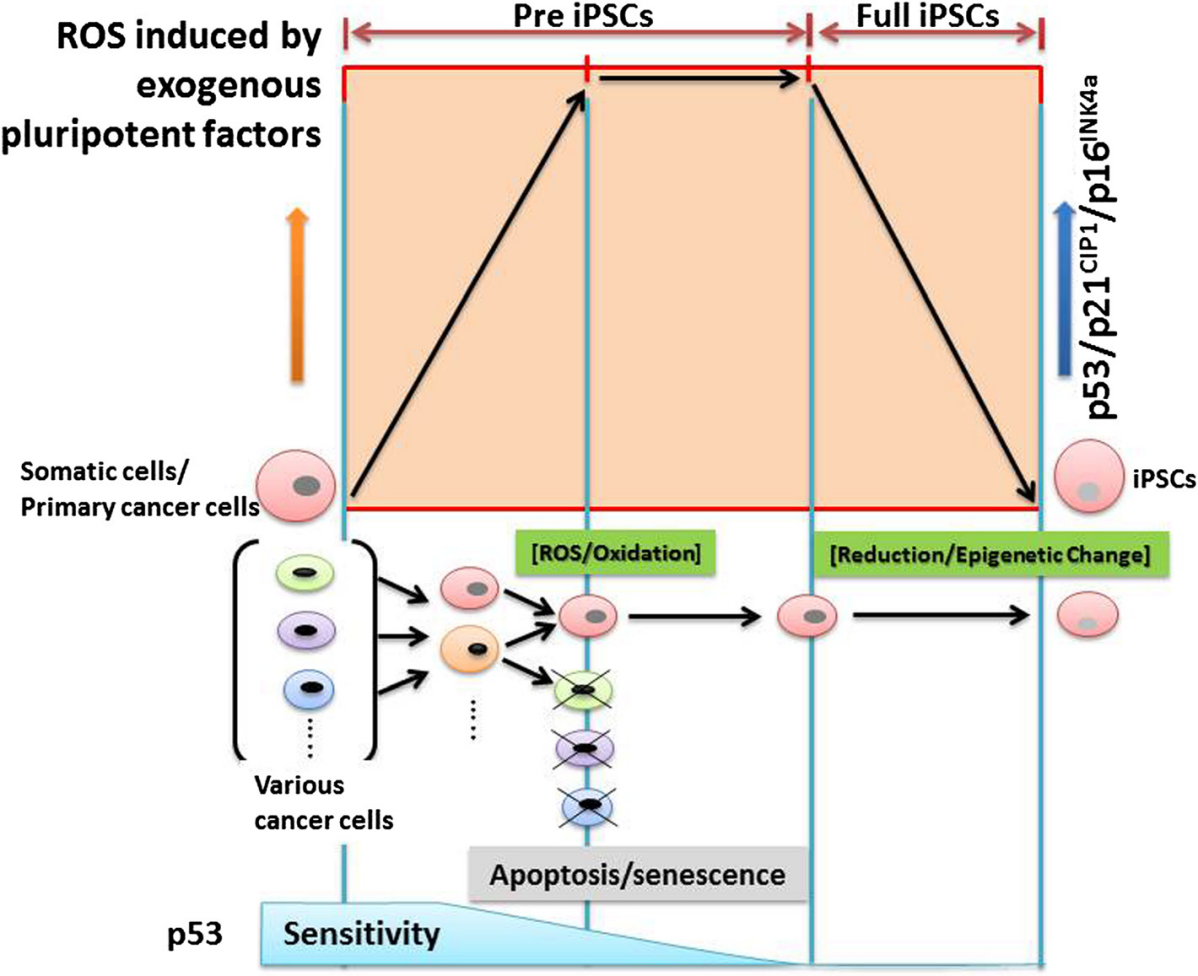
**Schematic representation of the nuclear reprogramming process from somatic cells, including primary cancer cells.** The initial stage of reprogramming includes the induction of somatic cells to pre-induced pluripotent stem cells (Pre-iPSCs) by exogenous pluripotent factors (such as Yamanaka 4 factors) via reprogramming-induced senescence (RIS; which results from DNA damage and metabolic stresses), which results in expression of tumor suppressor genes (such as *p21*^*CIP1*^ and p16^INK4a^) via the activation of p53. The subsequent process is triggered to overcome the barrier of RIS, cell apoptosis, or cell-cycle arrest by shutting off the function of tumor suppressor genes such as *p16*^*INK4a*^, *p21*^*CIP1*^, and *p53*, and then inducing the full commitment of iPSCs (Full-iPSCs) by endogenous stemness genes, as described in the text. Thus, the reaction oxygen species (ROS) produced by oxidative stress might be critical for the induction of endogenous reprogramming factor genes through at least epigenetic changes or antioxidation reactions [60,69].

In addition, it was reported that senescence impairs the reprogramming to iPSCs, and that reprogramming triggers a stress response of senescence at the initial stage [60]. In fact senescence is the irreversible arrest during the G1 transition of the cell cycle that is elicited by replicative exhaustion or in response to stresses such as DNA damage, drugs, or oncogenes. Moreover, oxidative stress also induces the cellular apoptosis and autophagy. These arrests are implemented primarily through the activation of p53 and the upregulation of the cyclin-dependent kinase inhibitors p16^INK4a^ and p21^CIP1^[61]. The introduction of Yamanaka factors initially triggers stress responses with characteristics of oxidative stress-like increases in the oxidized 8-oxoguanine and reprogramming-induced senescence (RIS) by upregulating p53, p16^INK4a^, and p21^CIP1^ at the initial stage (pre-induced pluripotent stem cells (pre-iPSCs)). This upregulation of p16^INK4a^ and p21^CIP1^ was observed in heterokaryon-based reprogramming [62], suggesting the existence of an inherent link between senescence and reprogramming. Subsequently, the elevated levels of p16^INK4a^ and p21^CIP1^ that were detected in pre-iPSCs were decreased at a later stage in mouse embryonic fibroblasts, and increased levels of p53 and p21^CIP1^ in IMR90 cells were also decreased at a later stage [60,63,64]. The inhibition of senescence using knockdown constructs of p53, p21^CIP1^, and p16^INK4a^ at the late stage finally improved the efficiency of the reprogramming of somatic cells or primary cancer cells, and the resulting iPSCs displayed characteristics of pluripotent stem cells (full-iPSCs) [60,65]. Other reports have confirmed the involvement of these two steps in reprogramming to full-iPSCs. Pre-iPSCs that failed to reprogram fully are trapped in a late step of reprogramming [63]. Inhibition of DNA methylation, knockdown of lineage-specific genes, or treatment with two inhibitors [66] can either convert some of these pre-iPSCs to full-iPSCs, or increase the proportion of fully reprogrammed iPSCs versus pre-iPSCs. The inhibition or the alleviation of senescence can increase the number of cells that surpass the early barrier imposed by RIS, resulting in a higher number of both pre-iPSCs and fully reprogrammed iPSCs. A combination of both strategies may be used synergistically to enhance reprogramming efficiency. RIS and probably reprogramming-induced apoptosis act as an initial barrier that limits the efficiency of the reprogramming. The reprogramming is slower and stochastic, suggesting the existence of a barrier that limits its efficiency. To increase the efficiency of reprogramming, the repression of RIS or reprogramming-induced apoptosis is definitely required at the late stage followed by a decrease in the expression of p16^INK4a^, p21^CIP1^, and p53 by hypoxic or other conditions, which are necessary for full reprogramming [43,45-47,67,68] (Figure 1).

The tumor suppressor p53 has been studied most extensively as a pivotal signal that converts diverse upstream stresses into downstream responses, including cell-cycle arrest, senescence, DNA repair, reprogramming, and programmed cell death [70]. p53 has been implicated as an enforcer of differentiation by virtue of its ability to limit the stem cell characteristic of self-renewal in several systems [65,71]. Together with the demonstration by Yamanaka that differentiated cells can be reprogrammed to a dedifferentiated state [67], and the demonstration that p53 is a potent reprogramming barrier [43-47,53,68], this has led to a resurgence of interest in the idea that loss of differentiation [72] may be linked to p53 pathway disruption in tumors. Recent studies have provided additional evidence of the link between p53 and the emergence of dedifferentiated, stem-like phenotypes [73]. The implications of these findings are far-reaching and will cause us to reconsider the role played by p53 inactivation in tumor pathophysiology and, more generally, the relationship between stem cells and cancer. Thus, reprogramming requires two stages: the initial stage includes ROS production induced by reprogramming factors, which leads to the reprogramming changes or DNA damage that induce the expression of p16^INK4a^, p21^CIP1^ and p53. At this late stage, these alterations should be shut down by reducing expression of p53, p21^CIP^, and p16^INK4a^ via hypoxic conditions or the expression of stemness genes such as *OCT4*, *SOX2*, *NANOG*, or other pluripotent genes (Figure 1).

The efficacy of reprogramming is indeed increased by several fold, but these iPSCs reprogrammed from cancer cells sometimes maintain or produce p53 mutations, resulting in tumor formation. Several genes in the original Yamanaka iPSC cocktail, such as c-MYC, generate oncogenic stresses that activate the p53 pathway to induce cell-cycle arrest or death [74]. Consequently, c-MYC expression, together with general tissue culture stresses, would be expected to activate p53 during the generation of iPSCs, to reduce reprogramming frequency or rate. These results have several important implications. First, subtle changes in p53 activity are all that is needed to increase the probability of reprogramming. At initial stages, the reprogramming factors induce ROS production by DNA damage and repair function and, at a later stage, these ROS should be suppressed by the antioxidation system of cells or other epigenetic changes [75]. At this stage, p53-related pathways are required for epigenetic chromatin changes. Second, reprogramming is limited by a variety of p53-induced protective pathways during the late stage. Finally, through its ability to inhibit cell-cycle progression, p53 provides a potent barrier to the acquisition of the epigenetic changes that underlie the dedifferentiation involved in iPSC formation. Thus, p53-dependent pathways are required to inhibit the mutation of cells at the initial stage. Subsequently, to overcome p53-dependent senescence, cell-cycle arrest, and apoptosis, p53 downregulation by epigenetic reprogramming via the induction of stemness genes is required. A controllable system that was used to analyze reprogramming kinetics indicated that p53 inhibition enhances the generation of iPSCs probably through cell-cycle acceleration [76], although the data did not exclude the possible involvement of cell-cycle-independent contributions.

### Reduction of the risk of tumorigenicity during the reprogramming of induced pluripotent stem cells

**Figure 2:**
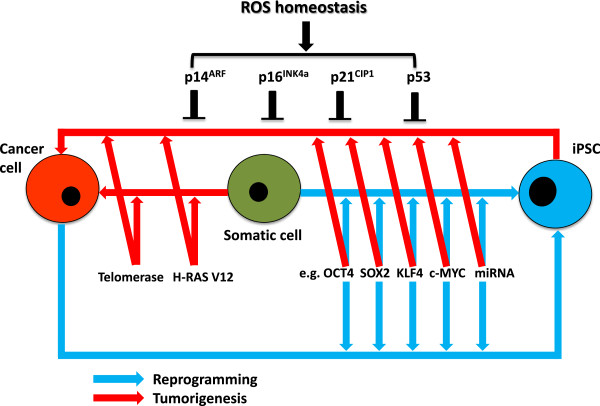
**Schematic diagram of the overlapping mechanisms between cellular reprogramming and tumorigenesis.** Overexpression of pluripotency factors (such as OCT4, SOX2, KLF4, c-MYC, and microRNAs) and inhibition of tumor suppressor gene products (such as p14^ARF^, p16^INK4a^, p21^CIP1^, and p53) drive the generation of pluripotency (blue arrows) and tumorigenicity (red arrows) in the presence of activated telomerase [77] and H-Ras V12 [25] genes. These tumor suppressor genes are hypermethylated and silenced during the reprogramming and tumorigenic processes. iPSC, induced pluripotent stem cell; ROS, reactive oxygen species.

New cancer therapies based on the reprogramming approach using oncogenic pluripotency factors might increase the risk of tumor formation. Therapies that enhance the expression of tumor suppressor genes such as *p53*, *p16*^*INK4a*^, *p14*^*ARF*^ and *p21*^*CIP1*^, accompanied by at least one pluripotency factor (OCT4 or SOX2) used with a plasmid-delivery system to target cancer cells, seem more advantageous. This combination inhibits ROS production first and reduces the expression of tumor suppressor genes via the induction of endogenous stemness genes. This method of iPSC generation is efficient and effective without any mutation of tumor suppressor genes, thus resulting in the generation of normal, non-mutated iPSCs (Figure 2).

It is commonly recognized in the field of stem cell research and regenerative medicine that tumorigenic risks must be overcome before the start of human iPSC-based clinical applications. Several possible risks need to be avoided: (i) the prolonged adaptation of human ESCs in culture conditions often results in gains of chromosomes 12 and 17 [78-80] and iPSCs [81], which might induce tumor formation; (ii) iPSCs derived from normally discarded human placental tissues, such as the amnion, chorion, umbilical cord, or fetal blood, might minimize the acquisition of genetic and epigenetic alterations, and, therefore, might be safer than iPSCs derived from adult somatic cells or cancer cells [82,83] (Table 1). Cultured human amniotic membrane-derived cells can differentiate into cells of all three germ layers under both *in vitro* and *in vivo* conditions [84,85]. In fact, primary amniotic tissues have low immunogenicity and anti-inflammatory properties [86,87], and the expression of putative immunosuppressive factors, such as CD59 and CD73, is lost during the reprogramming process [88]. The loss of those factors in reprogrammed human amniotic membrane-derived iPSCs might benefit their potential therapeutic application. Upregulated CD44 expression may be a surrogate marker of p53 inactivation and associated plasticity; thus, we will screen for the risk of developing tumorigenicity using this CD44 marker [89]. These approaches to avoid the cause of tumorigenicity might be useful when we treat stem cells or iPSCs with cancer-inducing agents, or when generating full reprogramming stem cells from somatic cells.

**Table 1:** Characteristics of various types of pluripotent stem cells

**Stem cell type**	**Donor cell type**	**Reprogramming factors/systems**	**Teratoma formation**	**Chimera formation**	**Pluripotency marker expression**			**Reference**
					**SSEA1**	**SSEA3/4**	**OCT4,SOX2, NANOG**	
MESCs	Embryo	-	+	+	+	-	+	[9]
HESCs	Embryo	-	+	ND	-	+	+	[7]
MEpiSCs	Epiblast	-	+	-	+	-	+	[90]
MiPSCs	Neural stem cell	OCT4, viral	+	+	+	_	+	[91]
HiPSCs	Amnion	SOX2, plasmid electroporation	+	ND	-	+	+	[82]
MiPSCs-C	Melanoma	OCT4, c-MYC, KLF4, viral	+	+	ND	ND	ND	[43]
HiPSCs-C	Colorectal cancer	OCT4, c-MYC, SOX2, KLF4, viral	+	ND	-	+	+	[92]
HiPSCs-C	Melanoma	miRNA, viral	+	ND	-	+	+	[93]

Human and murine pluripotent stem cells are characterized as described in the cited references. HESCs, human embryonic stem cells; HiPSCs, human induced pluripotent stem cells; HiPSCs-C, human induced pluripotent stem cells from cancer; MEpiSCs, murine epiblast stem cells; MESCs, murine embryonic stem cells; MiPSCs, murine induced pluripotent stem cells; MiPSCs-C, murine induced pluripotent stem cells from cancer; ND, not determined.

### Merits of reprogrammed cancer cells as a cancer model

These reprogrammed cancer cells from cancer patients may alternatively be used to find genetic and epigenetic clues as to how the nuclear reprogramming was blocked when generating fully competent iPSCs or stem cells. Indeed, reprogrammed cancer cells generated from patients for the induction of pluripotent cells provided a potential cell-based therapy model to restore tissues or organs destroyed by chemotherapy, even though these cells are not fully pluripotent cells [89]. Accumulating evidence indicates that the epigenetic mechanism affects the properties of reprogrammed iPSCs, and appears to retain epigenetic imprinting associated with their tissue type of origin [94]. Thus, epigenetic mechanisms have been recognized to play important roles in cancer development and cell differentiation. Based on these results, the reprogrammed cancer cells can serve as the ideal model system to study the molecular mechanisms of tumorigenesis and the properties of cancer stem cells to establish critical approaches for cancer and regenerative medicine.

## Conclusion

Here we have reviewed the tumorigenicity risks associated with iPSCs. Recently, genetic alterations, including copy-number variations and protein-coding point mutations, were observed during the reprogramming process by using high-resolution genetic approaches [95,96]. Point mutations were enriched in cancer-related genes [95]. These studies strongly suggest iPSCs have a high tumorigenicity potential. Thus, specifically, to achieve the therapeutic application of cancer cells via the reprogramming method, transfection of tumor suppressor genes, such as *p16*^*INK4a*^/*RB*, *p21*^*CIP1*^, *p14*^*ARF*^ and *p53*, combined with pluripotent factors, such as OCT4 or SOX2, might be preferable compared with viral transduction of potent oncogenes. Importantly, reprogramming and senescence are related processes, as shown by studies demonstrating that the reprogramming of cells is more challenging in cells that are closer to the onset of senescence [69]. The expression of reprogramming factors triggers RIS by activating several tumor-suppressive mechanisms. In addition, gene expression profiling studies have revealed that signature genes that are activated during reprogramming are common to these antiproliferative responses [69]. The small number of reports on the reprogramming of human primary cancer cells limits our ability to decipher the biological or technical barriers that prevent the reprogramming of cancer cells. However, we emphasize that human pluripotent stem cells should be checked to eliminate the possibility of any mutations in tumor suppressor genes, as they may lead to tumorigenesis after transfer to patients.

## Abbreviations

ESC: embryonic stem cell; full-iPSC: fully committed induced pluripotent stem cell; iPSC: induced pluripotent stem cell; mTOR: mammalian target of rapamycin; pre-iPSC: pre-induced pluripotent stem cell; RIS: reprogramming-induced senescence; ROS: reactive oxygen species.

## Competing interests

The authors declare that they have no competing interests.

## Authors’ contributions

Y-CL and C-SL contributed to study conception, manuscript writing and data analysis. YM, KH and YN critically revised the paper. Y-CL, KKY and SS contributed to study conception, data analysis, and manuscript writing and critically revised the paper. All authors read and approved the final manuscript.
